# Dataset on significant risk factors for Type 1 Diabetes: A Bangladeshi perspective

**DOI:** 10.1016/j.dib.2018.10.018

**Published:** 2018-10-09

**Authors:** Sayed Asaduzzaman, Fuyad Al Masud, Touhid Bhuiyan, Kawsar Ahmed, Bikash Kumar Paul, S.A.M. Matiur Rahman

**Affiliations:** a Department of Software Engineering (SWE), Daffodil International University, Dhaka, Bangladesh; b Department of Information and Communication Technology (ICT), Mawlana Bhashani Science and Technology University, Tangail, Bangladesh

**Keywords:** Dataset on Type-1 Diabetes, Analysis of data, Bangladesh perspective, Data of significant factors

## Abstract

In this article, dataset and detailed data analysis results of Type-1 Diabetes has been given. Now-a-days Type-1 Diabetes is an appalling disease in Bangladesh. Total 306 person data (Case group- 152 and Control Group- 154) has been collected from Dhaka based on a specific questioner. The questioner includes 22 factors which were extracted by research studies. The association and significance level of factors has been elicited by using Data mining and Statistical Approach and shown in the Tables of this article. Moreover, parametric probability along with decision tree has been formed to show the effectiveness of the data was provided. The data can be used for future work like risk prediction and specific functioning on Type-1 Diabetes.

**Specifications table**

**Table:** 

Subject area	*Biology*
More specific subject area	*Significant Risk Factors analysis from Data of Type 1 Diabetes using Statistical and Data Mining Approach.*
Type of data	*Table, figure, Raw Dataset*
How data was acquired	*Survey, Questioner*
Data format	*Raw, analyzed*
Data source location	*From different hospitals and diagnostic center in Dhaka, Bangladesh.*
Data accessibility	*Data is within this article*

**Value of the data**

• This data can be used at research in Type-1 Diabetes for Bangladeshi perspective. The size of data can be extended by the factors in which data is collected
• Provided data can be used in not only significance analysis but also in risk prediction functioning.
• These data introduced new approach of risk factor prediction and finding the significance level among factors as well as sub factors.
• Analyzed Dataset of both Data Mining and Statistical approach illustrates the comparison effect and realistic outcome of the research.

## 1 Data

Data provided in this article based on different factors among Type-1 Diabetes. Table 1, Table 2 Table 3 and Table 4 shows the significance level of Factors according to Info Gain, Gain Ratio, Gini Index and Chi-square (χ^2^)– Test. Table 1 illustrates the significance among the factors according to the analysis whereas Table 2, Table 3 and Table 4 also shows the significance level of sub factors like (Symptoms, Family history of Type-1 and Type-2 Diabetes). Table 5 shows the key factors on data analysis. Table 6 shows the Correlation among the significant factors which describes the dependency among the factors. P values and 95% C.I is shown in Table 7 which shows the significant factors. The factors whose P value is > 0.05 is significant and is shown in the table. Table 8 depicts the probability of Type-1 Diabetes according to data. The probability are shown among the factors and sub factors which leads to conclude effectiveness of those sub factors in Type-1 Diabetes.

**Table 1:** Data table on significance of factors according to Info Gain, Gain Ratio, Gini Index and χ^2^-test.

**Rank**	**Factors**	**Info. gain**	**Gain ration**	**Gini**	**χ**^**2**^**- Test**
1	HbA1c	0.520	0.522	0.284	111.447
2	Hypoglycemia	0.464	0.506	0.253	103.342
3	Age	0.286	0.154	0.179	92.146
4	Pancreatic disease affected in child	0.321	0.386	0.167	77.000
5	Area of Residence	0.210	0.136	0.136	45.003
6	Education of Mother	0.123	0.129	0.082	18.491
7	Adequate Nutrition	0.157	0.187	0.100	16.361
8	Autoantibodies	0.243	0.334	0.129	15.961
9	Sex	0.061	0.061	0.041	11.843
10	Family History affected in Type-1 Diabetes	0.031	0.035	0.021	9.081
11	Family History affected in Type-2 Diabetes	0.019	0.019	0.013	4.434
12	Standardized growth rate infancy	0.054	0.074	0.033	2.741
13	Standardized birth weight	0.096	0.122	0.052	0.517
14	Impaired glucose metabolism	0.001	0.001	0.000	0.226

**Table 2:** Data table on significance of factors according to Info Gain, Gain Ratio, Gini Index and χ^2^-test (family history in Type-1 Diabetes).

**Family History in Type-1 Diabetes**	**Info. gain**	**Gain ratio**	**Gini**	**χ**^**2**^-**Test**
Mother	0.026	0.058	0.017	9.354
Father׳s Heredity	0.022	0.047	0.015	8.211
Mother׳s Heredity	0.006	0.012	0.004	2.309
Father	0.001	0.004	0.001	0.514

**Table 3:** Data table on significance of factors according to Info Gain, Gain Ratio, Gini Index and χ^2^-Test (family history in Type-2 Diabetes).

**Family History in Type-2 Diabetes**	**Info. gain**	**Gain ratio**	**Gini**	**χ**^**2**^-**Test**
Mother	0.033	0.089	0.021	11.847
Father׳s Heredity	0.007	0.009	0.005	2.217
Father	0.003	0.005	0.002	1.027
Mother׳s Heredity	0.001	0.001	0.001	0.290

**Table 4:** Data table on significance of factors according to Info Gain, Gain Ratio, Gini Index and χ^2^-Test (different symptoms).

**Symptoms**	**Info. gain**	**Gain ratio**	**Gini**	**χ**^**2**^**-Test**
Frequent Urination	0.668	0.681	0.364	129.684
Increased thirst	0.668	0.681	0.364	129.684
Fatigue and Weakness	0.573	0.597	0.314	118.539
Unintended weight loss	0.505	0.540	0.276	109.421
Extreme Hunger	0.445	0.490	0.242	100.303

**Table 5:** Comparative result dataset of factors using different algorithms.

**Ranker Algorithm**	**BestFirst / Greedy Stepwise Algorithm**
HbA1c	Age
Hypoglycemia	Sex
pancreatic disease affected in child	Area of Residence
Age	HbA1c
Autoantibodies	Adequate Nutrition
Area of Residence	Standardized growth-rate in infancy
Adequate Nutrition	Autoantibodies
Education of Mother	Family History affected in Type 1 Diabetes
Standardized birth weight	Hypoglycemis
Sex	pancreatic disease affected in child
Standardized growth-rate in infancy	N/A
Family History affected in Type 1 Diabetes	N/A
Family History affected in Type 2 Diabetes	N/A
Impaired glucose metabolism	N/A

**Table 6:** Correlation data among factors using Apriori Algorithm.

**No**	**Correlation**
1	Standardized growth-rate in infancy (Middle quartiles pancreatic disease affected in child) **==>** Standardized birth weight Middle quartiles
2	Autoantibodies pancreatic disease affected in child ==> Standardized birth weight Middle quartile
3	Adequate Nutrition (Yes)- Standardized growth-rate in infancy (Middle quartiles) ==> Standardized birth weight (Middle quartiles)
4	pancreatic disease affected in child =No 230 ==> Standardized birth weight=Middle quartiles 217 <conf:(0.94)> lift:(1.09) lev:(0.06) [18] conv:(2.25)
5	Adequate Nutrition (Yes) ==> Standardized birth weight (Middle quartiles)
6	Hypoglycemis (No) ==> Standardized birth weight (Middle quartiles)
7	. Hypoglycemis (No) ==> pancreatic disease affected in child (No)
8	Standardized growth-rate in infancy (Middle quartiles) Autoantibodies (Yes) ==> Standardized birth weight (Middle quartiles)
9	Hypoglycemis ==> Autoantibodies
10	Standardized growth-rate in infancy (Middle quartiles) Impaired glucose metabolism==> Standardized birth weight (Middle quartiles)

**Table 7:** P value and confidence interval of risk factors in Type-1 Diabetes dataset.

**Factors**	**P-value**	**95% C. I for Odds ratio**	
		**Lower**	**Upper**
Age	0.000*	0.2633	0.4884
Less than 5			
Less than 11			
Less than 15			
Greater than 15			
Sex	0.000*	0.1111	0.2235
Male			
Female			
Area of Residence	0.000*	0.1489	0.3162
Rural			
Urban			
Suburban			
Height	0.665	0.245	0.0384
Weight	0.996	1.88	0.1.89
BMI	0.996	0.70	0.70
Adequate Nutrition	0.008	0.0173	0.1163
Yes			
No			
Education of Mother	0.999	0.0544	0.0544
Yes			
No			
Standardized growth-rate infancy	0.999	0.251	0.251
Lowest quartile			
Middle quartile			
Highest quartile			
Family History in Type-1 Diabetes	0.000*	0.4522	0.5550
Father			
Mother			
Father׳s Heredity			
Mother׳s Heredity			
Family History in Type-2 Diabetes	0.000*	0.1864	0.2986
Father			
Mother			
Father׳s Heredity			
Mother׳s Heredity			

* Significant Factors

**Table 8:** Data for probabilities and effectiveness of factors in Type-1 Diabetes.

**No**	**Factors**	**Subfactors**	**Probabilities**	**Effectiveness**
1	Age	Greater then 15	0.88	High
		Less Than 15	0.42	Moderate
		Less than 11	0.2	Low
		Less than 5	0.18	Very Low
2	HBA1c	Less than 7.5	0.21	Low
		Greater than 7.5	0.72	High
3	Hypoglycemis	Yes	0.69	High
		No	0.27	Low
4	Pancreatic Diseases diagnosed in affected childs	Yes	0.5	Moderate
		No	0.31	Low
5	Area of Residence	Rural	0.82	High
		Suburban	0.65	Moderate
		Urban	0.22	Low
6	Adequate Nutrition	No	0.86	High
		Yes	0.36	Low
7	Autoantibodies	No	0.4	Moderate
		Yes	0.38	Moderate
8	Sex	Female	0.65	High
		Male	0.36	Low
9	Family History type 1 Diabetes	Yes	0.68	High
		No	0.41	Low
10	Family History type 2 Diabetes	Yes	0.59	High
		No	0.44	Low
11	Standard Growth Rate	Lowest	0.96	High
		Height	0.72	Moderate
		Middle	0.45	Low

## 2 Methodology of data analysis

Type 1 Diabetes is now a concerning factor that is increasing at an alarming rate in low incoming country like Bangladesh. The increase in Blood glucose level (Hypoglycemia) causes Type-1 Diabetes in childhood [1]. Work on dataset of Type-1 Diabetes [2] in different regions of the world has been done in recent years [3]. In this paper, dataset on Type-1 Diabetes has been provided for Low incoming country like Bangladesh.

### 2.1 Data collection and preprocessing

Data of Type-1 Diabetes was collected from Different Hospitals and Diagnostic center from Dhaka, Bangladesh. The Data collection process was done by following a questioner. The questioners have been formed by previous research studies and discussion with medical persons. Both Case (Affected) and Control (Unaffected) group data was collected for both male and female. The total data size is 306 where 152 was affected (Case) and 154 was unaffected (control) groups. The total 22 Factors (like Age, Sex, Area of residence, Education of Mother, Hba1c, BMI) was considered in account to collect fruitful data.

After data collection there may be some inconsistent, missing and uncategorized data. Data preprocessing or so called data cleaning has been done using a Data preprocessing Feature of WEKA (A data Mining Tool). In previous studies [4] data is also preprocessed for future action.

### 2.2 Data mining approach

To find significant factors two Data mining tools Orange and WEKA was used. Probability of sub factors, χ^2^-Test, Info gain etc was done by Orange. WEKA was used for algorithm based analysis. WEKA was also used to find correlation among the factors using Apriori Algorithm. By these procedures the significance level among the factors are explored on the Dataset.

### 2.3 Statistical approach

Statistical approach has been used to find significance and correlation in article [5]. We have used SPSS V20.0 to find out the P-Value and Confidence Interval. By P value the significant factors can easily be defined from the dataset.

### 2.4 Significance formulation

Factors like Hypoglycemia (increase glucose level) and Insulin are key factors for Type-1 Diabetes [6], [7]. By all the data and Tables from the dataset the final decision tree can be formed. By the decision tree we can easily describe whether one person is affected or not.

**Fig. 1:**
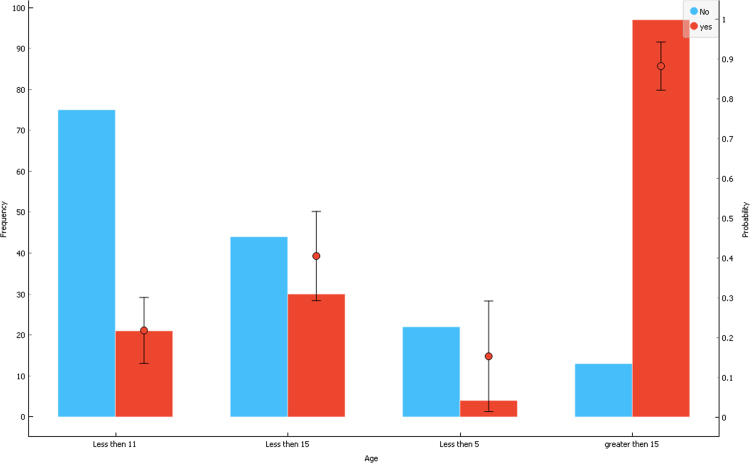
Data on 2-D view of probability distribution of the age with respect to affected group.

**Fig. 2:**
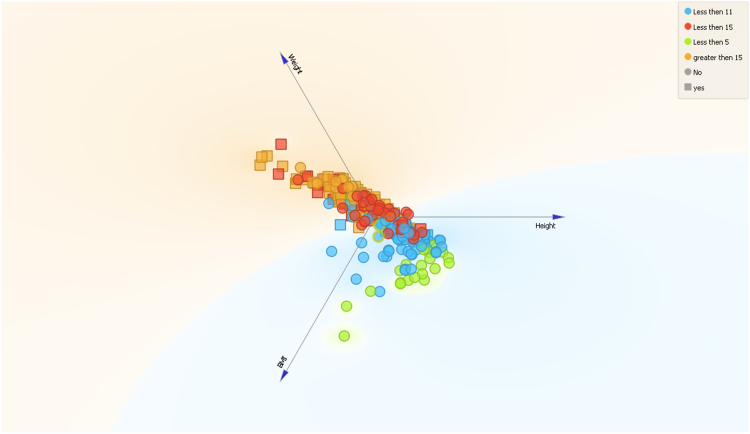
3-D visualization of the analyzed dataset and data distribution for BMI, height and weight.

**Fig. 3:**
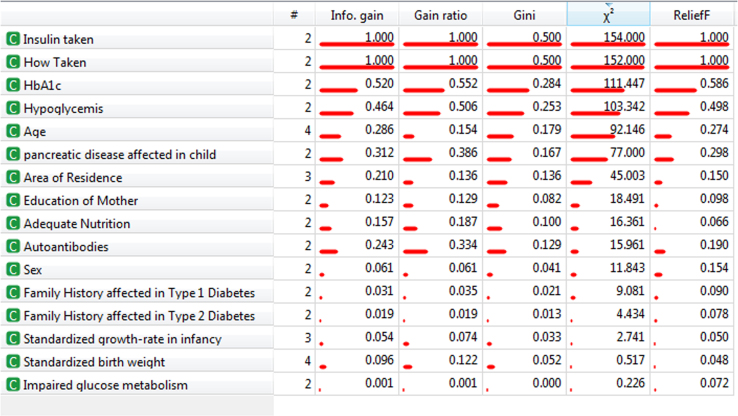
Visualization of parameters and its outcomes of dataset.

**Fig. 4:**
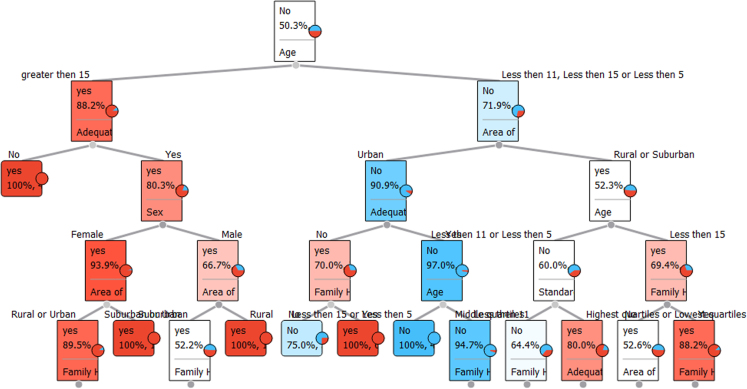
Decision tree among the factors of Type-1 Diabetes.

Disease Risk prediction and its analysis on dataset for different disease has been done before by Ahmed et al. in [8]. Fig. 1, Fig. 2, Fig. 3, Fig. 4 shows the detailed analysis results of data. The analysis was done using WEKA and Orange two different and powerful Algorithm based Data Mining Software. The outcome results and its data shows the risk factors and its significance to detect Type 1 Diabetes.

## Financial support

There is no financial support for this research.
