# Supplementary material for: Dataset on significant risk factors for Type 1 Diabetes: A Bangladeshi perspective
Source: Data Brief. 2018 Oct 9;21:700–8. doi: 10.1016/j.dib.2018.10.018 (PMC6205358; doi:10.1016/j.dib.2018.10.018)
Supplement: Supplementary file 1 — Supplementary material [file mmc1.docx]

**Declaration of Conflict of Interest**

14^th^ August, 2018

Editor In Chief

**Journal of Data in Brief**

Dear Sir,

The Authors of this article are Sayed Asaduzzaman , Fuyad Al Masud , Touhid Bhuiyan , Kawsar Ahmed , Bikash Kumar Paul , S A M Matiur Rahman. Among them Sayed Asaduzzaman and Kawsar Ahmed are from same institution Department of Information and Communication Technology (ICT), Mawlana Bhashani Science and Technology University, Tangail, Bangladesh. Sayed Asaduzzaman is presently a Masters Student and Kawsar Ahmed is a Faculty. Fuyad Al Masud , Touhid Bhuiyan, Bikash Kumar Paul and S A M Matiur Rahman are from Department of Software Engineering (SWE) , Daffodil International University , Dhaka, Bangladesh. Among them Fuyad Al Masud is a Student and Touhid Bhuiyan, Bikash Kumar Paul and SAM Matiur Rahman are Faculties. The authors are fully institutional and are not involved in elsewhere. All the authors Declares no conflict of interest among them.

………………………………………………………………..

Best Regards

Sayed Asaduzzaman

Department of Information and Communication Technology (ICT)

Mawlana Bhashani Science and Technology University,

Tangail , Bangaldesh-1902.
