# Supplementary material for: Dataset on significant risk factors for Type 1 Diabetes: A Bangladeshi perspective
Source: Data Brief. 2018 Oct 9;21:700–8. doi: 10.1016/j.dib.2018.10.018 (PMC6205358; doi:10.1016/j.dib.2018.10.018)
Supplement: Supplementary file 2 — Supplementary material [file mmc2.zip › Detailed Dataset/Final Questioneer .docx]

Daffodil International University

Department of Software Engineering

| 1. Age of Children 2. <=5 years 3. <=11 years 4. >15 years   2. Sex  a. Male  b. Female   1. Area of Residence 2. Rural 3. Urban 4. Suburban 5. Duration of disease: 6. Weight: 7. Current BMI? 8. HbA1c:   a. Less than 7.5%  b. Over 7.5%  8. Adequate Nutrition  a. Yes  b. No  9. Education of Mother:  a. Yes  b. No  10. Standardized growth-rate in infancy:  a. Lowest quartile·  b. Middle quartiles  c. Highest quartile  d. Unknown | 11. Standardized birth weight:  a. Lowest quartile  b. Middle quartiles  c. Highest quartile  d. Unknown  12.Impaired glucose metabolism :  13. Autoantibodies :  14. Insulin taken  a. yes  b. no  If yes then How   1. Pump 2. Injection   15. Family History (Is there anybody in family affected in Type 1 Diabetes)  a. Father  b. Mother  C. Father’s Heredity  d. Mother’s Heredity  16. Is there anybody affected in type 2 diabetes?  a. Father  b. Mother  C. Father’s Heredity  d. Mother’s Heredity  17. Hypoglycemis?  a. yes  b. no  18. pancreatic disease diagnosed in the affected child s  a. Yes  b. No  19.Other Disease:  20.Symptoms:  a. Increased thirst  b. Frequent urination  c. Extreme hunger  d.Unintended weight loss  e. Fatigue and weakness. |
| --- | --- |
